# Estimating the Location and Spatial Extent of a Covert Anthrax Release

**DOI:** 10.1371/journal.pcbi.1000356

**Published:** 2009-04-10

**Authors:** Judith Legrand, Joseph R. Egan, Ian M. Hall, Simon Cauchemez, Steve Leach, Neil M. Ferguson

**Affiliations:** 1 MRC Centre for Outbreak Analysis and Modelling, Department of Infectious Diseases Epidemiology, Imperial College London, London, United Kingdom; 2 Microbial Risk Assessment, Emergency Response Department, Centre for Emergency Preparedness and Response, Health Protection Agency, Wiltshire, United Kingdom; Johns Hopkins University, United States of America

## Abstract

Rapidly identifying the features of a covert release of an agent such as anthrax could help to inform the planning of public health mitigation strategies. Previous studies have sought to estimate the time and size of a bioterror attack based on the symptomatic onset dates of early cases. We extend the scope of these methods by proposing a method for characterizing the time, strength, and also the location of an aerosolized pathogen release. A back-calculation method is developed allowing the characterization of the release based on the data on the first few observed cases of the subsequent outbreak, meteorological data, population densities, and data on population travel patterns. We evaluate this method on small simulated anthrax outbreaks (about 25–35 cases) and show that it could date and localize a release after a few cases have been observed, although misspecifications of the spore dispersion model, or the within-host dynamics model, on which the method relies can bias the estimates. Our method could also provide an estimate of the outbreak's geographical extent and, as a consequence, could help to identify populations at risk and, therefore, requiring prophylactic treatment. Our analysis demonstrates that while estimates based on the first ten or 15 observed cases were more accurate and less sensitive to model misspecifications than those based on five cases, overall mortality is minimized by targeting prophylactic treatment early on the basis of estimates made using data on the first five cases. The method we propose could provide early estimates of the time, strength, and location of an aerosolized anthrax release and the geographical extent of the subsequent outbreak. In addition, estimates of release features could be used to parameterize more detailed models allowing the simulation of control strategies and intervention logistics.

## Author Summary

Releasing highly pathogenic organisms into an urban population is a form of bioterrorism that could result in a large number of casualties. The first indication that a covert open-air release has occurred is quite likely to be individuals reporting for medical attention. If such an attack is suspected, then public health authorities would attempt to identify those individuals who have been infected in order to provide rapid treatment with the aim of reducing the possibility of disease and potential death. Aiming treatment at too small an area might miss individuals infected further down and/or up wind, whereas issues surrounding both treatment resources and serious side effects may rule out mass treatment campaigns of large sections of the population. Our work provides scientific robustness to firstly estimate where and when an aerosolized release has occurred and secondly identify the most critically affected geographic areas. In order to use this statistical tool during an outbreak, public health workers would only need to collect the time of symptomatic onset and the home and work locations of early cases; recent weather information would also be required. Although the accuracy of the estimates is likely to improve as more cases appear, treating individuals based on early estimates might prove more beneficial since time would be of the essence.

## Introduction

If clinical cases of anthrax were detected, public health decision makers would want to estimate as soon as possible the features of the exposure event leading to the outbreak in order to determine who has potentially been exposed and should receive prophylaxis [1]. Relevant variables include the date of exposure and the geographical extent of the outbreak. For example, data from the US anthrax outbreak of 2001 have been retrospectively explored to estimate the date of exposure of cases and how large the outbreak would have been if exposed individuals had not been treated [2],[3]. Later, Walden and Kaplan proposed an alternative method to estimate the time and size of an anthrax outbreak a few days after the occurrence of the first case and tested it on simulated data [4].

While the 2001 anthrax cases had been exposed through the US postal service [1], if the exposure was due to an outdoor airborne release other information such as the release location and the potential exposed area might be inferred from the data on observed cases. The methods discussed above do not allow the release location or the geographical extent of exposure to be estimated as they do not consider the localization of cases or the size of potentially exposed populations. More recently, Hogan et al. proposed the Bayesian Aerosol Release Detector (BARD) allowing the estimation of posterior distributions of the location, strength and date of a release based on pre-diagnostic (syndromic) medical surveillance data and meteorological data [5]. They evaluated the ability of the method to detect anthrax outbreaks with syndromic surveillance data and showed that it was able to detect simulated outbreaks with over 900 pre-diagnosed cases but performed poorly for smaller outbreaks. So far, the ability of the BARD to characterize a detected release has not been evaluated.

In this paper, we develop and evaluate the performance of a back-calculation method to characterize a release from the observation of the first few cases, population densities, meteorological conditions and population movements such as commuting data. We considered that the causative agent would have been identified from the first few cases and that the incubation period distribution of the disease would be known. We also explore the potential of our tool to inform the planning of mitigation strategies.

As a case study, we investigate a simulated release of *Bacillus anthracis* (the causative agent of anthrax), given its prominence on risk lists of pathogens and potential to be used in aerosolized biological weapons [1],[6].

## Methods

### Probabilistic anthrax model

We developed a probabilistic model for an inhalational anthrax outbreak following an instantaneous point source release. This model has three components: 1) the dispersion of anthrax spores in the atmosphere; 2) the within-host dynamics of anthrax spores; 3) the spatio-temporal population dynamics. We did not take into account cutaneous or gastrointestinal forms of anthrax.

The airborne dispersion of anthrax spores following an instantaneous point source release was modeled using a puff model weighted by the viability of spore concentration [7],[8]; this quantifies the average spore concentration at any location and time. However, for practical reason, we assumed that the average spore concentration was uniform over relatively small distances that characterize the spatial unit of our back-calculation method, i.e. Great Britain (GB) administrative wards, and equal to the concentration given by the puff model at the ward centroid. For each individual, the inhaled dose depends on the breathing rate and the spore concentration at his/her work place (from 9 am to 7 pm) and his/her residence (from 7 pm to 9 am). Other parameters such as the size of particles [1] would impact the inhaled dose but were not taken into account in our analysis for the sake of simplicity.

The within-host dynamics model describes the biological processes of clearance, germination and growth of anthrax spores within a host and was adapted from published models [3],[9],[10]. However, the model we developed considers continuous exposure rather than just instantaneous exposure. Once anthrax spores are inhaled into the lung, they are ingested by macrophages and can be destroyed. Surviving spores may germinate and then replicate [1]. Assuming that symptoms occur when the number of germinated spores exceeds a given threshold [10], the probability of developing disease can be written as the convolution of the cumulative distribution function of the time from exposure to first germination *F*_1_ and the density function of the time from first germination to symptoms.

Finally, the dispersion model and the within-host dynamics model are integrated with population density and movement data to model the spatio-temporal dynamics of the outbreak. Full details of the model are provided in Text S1.

### Characterizing an anthrax release

We used a Markov Chain Monte Carlo sampling algorithm [11] to estimate the time T, height H, strength (log10(S) where S is the number of released spores) and location W of the release. The posterior distribution of the parameters is detailed in Text S1. Given the rapid decline of spore concentration over time, we considered that an individual's entire dose was inhaled at the time of the release rather than continuously from this date. Following [12],[13],[14], we relied on the profile likelihood of the 3-dimension parameter space 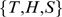:
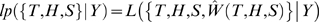
where *L*(.) is the likelihood function and Y are the observed data (dates of symptoms onset, residences and workplaces), both of which are presented in Text S1. 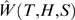 maximizes 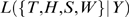 with respect to *W* and the parameter space 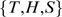 was explored with a standard Metropolis-Hastings algorithm.

### Evaluation of the method

To study the performance of our back-calculation method, we simulated 40 anthrax outbreaks due to a release at time *T* = 0 of strength *S* = 10^10^ spores in ward *W* = *W_0_* at height *H* = 100 m, using the probabilistic model described above. We used population and commuting data from the 1991 GB census for the 10,515 wards provided by the Office for National Statistics (see Text S1), the same meteorological stability conditions as Wein and colleagues used in a simulation study on the response planning to an anthrax attack [15], and parameter values provided in Table 1 [16]. Assuming that public health responses would ideally be initiated after only a few cases have been detected, the first 5, 10 or 15 cases developing symptoms were considered to have been observed. We then estimated the four parameters of the model characterizing the release (*T*, log_10_(*S*), *W*, *H*). The other parameters of the spore dispersion model and the within-host dynamics model embedded within the back-calculation were set at the literature-derived values used to generate the simulated data (see Table 1).

**Table 1:** Parameter Description and Values in the Reference Scenario.

Param.	Description	Units	Value in the ref. scenario	Ref
γ	Decay rate	/sec	1.67×10^−4^	[7]
λ	Germination rate	/day	1×10^−5^	[9]
θ	Clearance rate	/day	0.109	[9]
r	Growth rate	/day	11.7	*
b	Breathing rate	m^3^/min	0.03	[7]
k	Threshold for the number of bacilli before symptoms	bacilli	10^10^	[16]
	Median period between germination and symptoms	days	2	[9]
	Wind direction	BNG	(1,0)	
u	Wind speed	m/s	5.0	[15]
T	Date of the release	days	0	
S	Number of released spores	spores	10^10^	
H	Height of the release	m	100	[15]
W	Source	-	W_0_	

BNG: British National Grid System.

*: The growth rate was calibrated in order to have a median period between first germination and symptoms of 2 days according to equation (1.5) in Text S1.

We used medians of posterior distributions for height and strength estimates. The posterior distribution of the time was sometimes multimodal with local minima for night periods (we simulated a release during the day) and the median could fall into one of those local minima. Hence, to conserve the day/night information provided by the posterior distribution, instead of the time median, we discretized its posterior distribution into day/night classes and chose the middle time of the mode class as the point estimate. To estimate the release location, we also used the mode of the posterior distribution. Root mean square errors (RMSE) were used to summarize the quality of estimates (see definitions in Text S1).

In order to understand how misspecification of aspects of the model would impact estimation accuracy, we reproduced the estimation procedure but deliberately misspecified either parameter values, data or the model structure. We examined 5 scenarios (see Table 2):

**Table 2:** Description of Scenarios.

Scenario	Modified model/data	Misspecification type	Description
A	Symptoms onset dates of the simulated sample	Uncertainty on data	Onset dates precision = 0.5 days rather than 1 hour . For cases developing symptoms between 9AM and 9PM the registered time is 9 AM. For other cases the registered time is 9 PM.
B	Estimation	Parameter value of the within-host dynamics model	Median delay between germination and symptoms = 5 days rather than 2 days
C	Simulation	Within-host dynamics model	Incubation period for low doses given by [9] Instantaneous exposure
D	Simulation	Spore diffusion model	Spore concentration given by HPAC model [17] Instantaneous exposure
E	Simulation	Population movements	Occasional movements added to daily commuting data

- Reference scenario dataset used, but the precision of the symptoms onset date was 0.5 days rather than one hour. We considered that the symptoms onset hour of patients developing symptoms between 9AM and 9PM would be registered as 9AM, and 9PM for patients developing symptoms between 9 PM and 9 AM.
- Reference scenario dataset used, but median delay between germination and symptoms of 5 days assumed in the back-calculation model rather than the 2 days used to generate the data.
  In scenarios C to E, we simulated 40 outbreaks with three modified versions of our model and then used the reference scenario back-calculation model to fit these data:
- Modification of the within-host dynamics model. Datasets were generated using the reference scenario model but with the within-host dynamics component replaced by the model proposed by Brookmeyer *et al* for a low dose exposure [9]: the attack rate was computed as 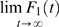 and the cumulative distribution function of the incubation period was 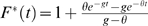 with *g* = 0.346 days^−1^ corresponding to a median delay between germination and symptoms of 2 days.
- Modification of the spore dispersion model. Datasets were generated using the reference scenario model but with the puff model of airborne dispersion replaced by the Hazard Prediction and Assessment Capability (HPAC) model [17]. The cumulative distribution function of the incubation period was the same as in the reference scenario but assumed an instantaneous exposure (see Text S1) and an attack rate given by 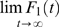.
- Modification of population movement assumptions. Instead of considering only commuting data, we considered that due to non-commuter travel, 10% of individuals could be exposed during the day in wards different from the ward where they would otherwise work. We considered that the pattern of these occasional movements was similar to the pattern of commuting movements. Hence, for 10% of cases, we considered that the original workplace was actually an occasional destination. The workplace of each of these cases was then drawn from the distribution of workplaces of people living in the individual's ward of residence. With this dataset, we also tested a modified version of the reference scenario back-calculation model by considering that people would have a small probability per day (set at 0.1) to travel away from their working place, with destinations being chosen based on ward sizes and distance to usual workplace (see Text S1 for details).

### Comparison with other back-calculation methods

Past studies [4],[18],[19] have sought to characterize an anthrax outbreak and, although they were not designed to estimate the release location, it is possible to compare the exposure date and outbreak size estimates they provide with our estimates. We ran our own versions of the Walden and Kaplan method [4] and the algorithm proposed by Ray et al [18] on the datasets generated with the reference scenario and scenarios C and D using the same incubation period distribution as in our algorithm. As the Walden and Kaplan method [4] assumes that the incubation period is not dose dependent, we used a low dose exposure (10 spores) although it should be noted that order of magnitude increases in the dose made little difference to the estimates (results not shown).

### Implication for mitigation policies

In terms of helping to plan mitigation strategies, the first issue we examined was whether our estimates would allow the prediction of the outbreak extent from data on the first few cases. We also examined whether the model could accurately infer the geographical extent of the outbreak, *i.e.* where and how many people had been exposed. Indeed, this could help to target interventions (such as prophylaxis and decontamination) at the most exposed populations for mitigation strategies and to assess the scale of effort (e.g. numbers of antibiotic courses) required. We considered a mitigation strategy whereby people living or working in a ward with a risk of being clinically infected greater than a given threshold (from 10^−5^ to 10^−8^) would be targeted for prophylactic treatment. The risk attributed to each ward was defined as the risk of developing disease following an exposure in this ward at the release time. We compared the model-inferred risk estimates (using risk posterior distribution medians) with the model-inferred risk values calculated with the real parameter values. To explore further the effectiveness of a targeted mitigation strategy based on the back-calculation model estimates, we determined how many cases would be prevented if all individuals exposed to a given risk according to our estimates received a 100% effective prophylactic treatment. We considered that the treatment would be administered 4 days after the 5^th^, 10^th^ or 15^th^ case had occurred to allow for a lag time between symptomatic onset of the last observed case and diagnosis, estimation, planning and implementation of interventions. In addition, treatment was assumed to prevent disease for all symptom-free individuals.

Finally, we compared the efficiency of the strategy described above with a “ring strategy” not requiring sophisticated analytical and computational methods. For this “ring strategy”, the wards considered at risk were located in the neighborhood of wards where the greatest number of cases had been detected (workplaces and residences were included). We selected as neighbors all wards having its centroid within a given distance of at least one of the centroids of the J most affected wards.

## Results

**Figure 1:**
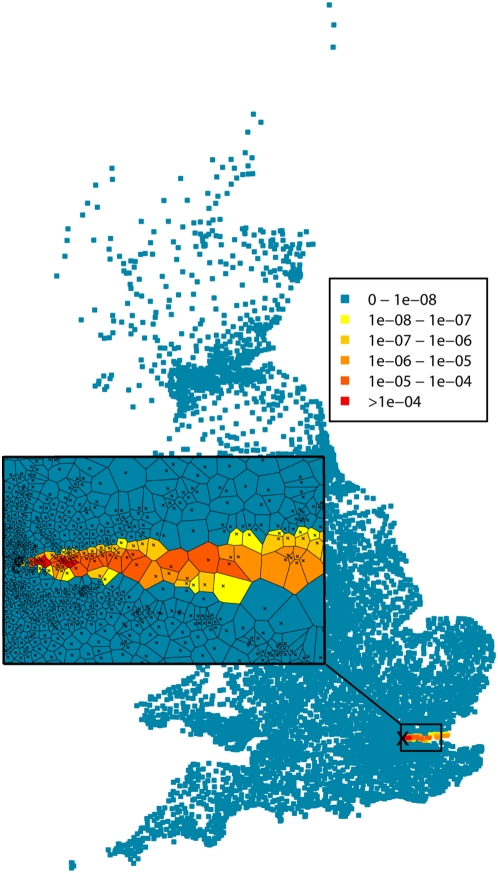
Map of the risk of anthrax infection (attack rates) in each ward for all scenarios except scenario D. The cross on the main map represents the location of all simulated releases. The inset map represents population-weighted ward centroids (crosses) and their Voronoi diagram (polygons).

Although we simulated outbreaks following a release in a populated area, the set of parameters we used lead to relatively small simulated outbreaks (average size = 27, range = 19–39, see the risk map in Figure 1 and the description of the simulated outbreaks in Text S1).

### Characterizing anthrax releases

**Figure 2:**
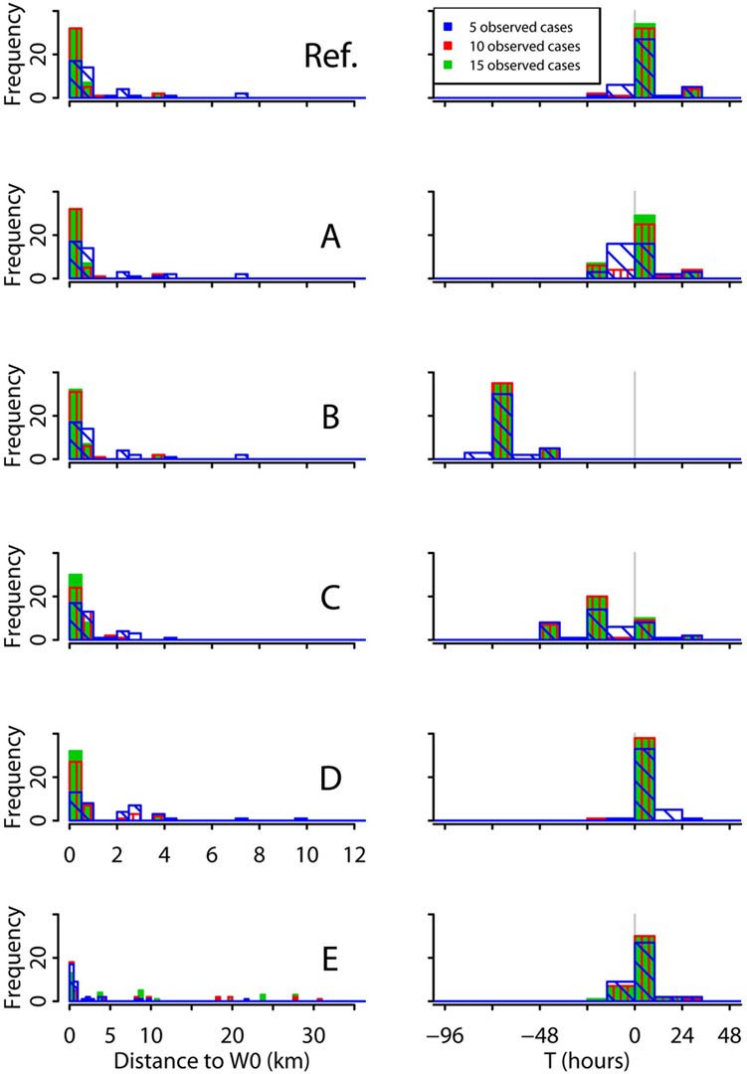
Histograms of the release location (left column) and date (right column) estimates for the 40 simulated outbreaks with Reference scenario (Ref.) and scenarios A to E. The release location is represented by the distance to the real source. For the date estimates, breaks were set at 9 AM and 7 PM and counts are represented by bar heights rather than bar surfaces. For two outbreaks of scenario D, the source location estimated with 5 observed cases was further than 12 kilometers (14.3 and 18.2 km). For scenario E, the source location estimated with 5 observed cases was further than 35 kilometers for two outbreaks (57 and 68 km), the source location estimated with 10 observed cases was further than 35 kilometers for one outbreak (57 km), the source location estimated with 15 observed cases was further than 35 kilometers for two outbreaks (45 km and 117 km).

Figure 2 (reference scenario) shows that we were able to localize and date the release with accuracy when using 10 cases. As shown in Text S1, using the median as date point estimates rather than the centre of the mode class gave similar results. Although decreasing the number of observed cases to 5 lowered the ability of the method to localize the release (real source identified in 17/40 outbreaks versus 32/40 with 10 observed cases), it was still able to date the release with accuracy (error<10 hours for 33/40 simulated outbreaks). Furthermore, the distance from the estimated source to the real source did not exceed 7.4 km with 5 observed cases and 3.8 km with 10 observed cases (average distance between workplace of cases ranges 3.7–14.0 km). The height of the release was more difficult to characterize and was correlated with the strength of the release (correlation of 0.78 on average). Bias could reach more than twice the real height and posterior distributions estimated with 5 observed cases were often flat (95% credible interval width was up to 1220 meters). An example of the 4 parameters posterior distributions estimated with data from 5, 10 or 15 observed cases is shown in Text S1. When comparing the estimated expected number of cases with the real expected number of cases, the root mean square relative error (see definition in Text S1) decreased from 70% for estimates based on 5 cases to 45% for estimates based on 10 cases (see Table 3).

**Table 3:** Performance of the Back-calculation Method to Fit the Expected Outbreak Size.

	Coverage* (%)			RMSE1 (%)			Error range**		
# observed cases	5	10	15	5	10	15	5	10	15
Reference	95	95	95	71	45	32	0–215	1–137	3–83
A‡	80	95	95	103	50	33	2–237	0–143	2–102
B‡	95	95	95	71	45	32	0–212	1–141	2–83
C‡	90	97.5	100	89	32	22	4–478	1–82	1–72
D‡	80	87.5	85	141	72	54	2–427	1–232	3–201
E‡	87.5	90	90	90	52	35	2–412	1–189	1–119

RMSE1 = Relative root mean square error (see definition in Text S1).

*: The coverage is defined as the probability that the real value falls in the (2.5^th^, 97.5^th^) percentiles interval of the posterior distribution.

**: Range of the absolute relative error (%).

**‡:** See Table 2 and Methods for description of scenarios A to E.

### Sensitivity analysis

With scenario A, although estimates of the timing of release were slightly modified (for estimates based on 5 cases, difference ranged 0–2 days), the bias was below 10 hours for 80% of the simulated outbreaks. The accuracy of the source location estimates was not affected (see Figure 2).

Similarly, increasing the median delay between spore germination and symptoms from 2 to 5 days in the estimation algorithm (scenario B) modified estimates of the time of release by 71 hours on average (compared to the reference scenario estimates) but it did not modify the performance of the method to characterize the release location. Misspecifying further the within-host dynamics model (scenario C) by simulating symptomatic onset dates with the incubation period distribution for low doses proposed by Brookmeyer and colleagues [9] affected the precision of the release date estimates (RMSE about 24 hours with scenario C versus 12 hours with the reference scenario) but the estimates of the other release features (location, height and strength) remained accurate (see Figure 2 and Text S1) .

When we used a different spore dispersion model (HPAC) to simulate outbreaks (scenario D), the source location estimates based on 5, 10 and 15 observed cases were somewhat (though not catastrophically) impaired (RMSE = 4.6, 1.2, 0.6 km respectively versus 2.0, 0.9, 0.7 km with the reference scenario). Release height and strength estimates were also biased (see Text S1) but the release date estimates remained accurate. Increasing the number of observed cases from 10 to 15 increased substantially the quality of the source location estimates whereas this wasn't the case for the reference scenario and scenarios A to C for which the RMSE of the source location estimates based on 10 observed cases were less than 1 km.

**Figure 3:**
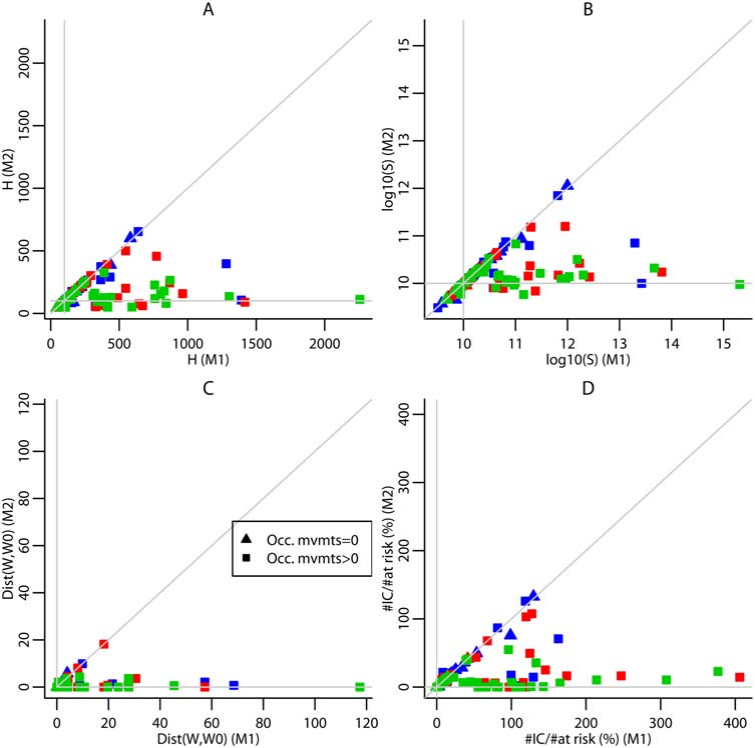
Comparison of the estimates based on the standard model (M1) with estimates based on the model allowing for occasional movements during the day (M2). Estimates of the height (A), strength (B) and location (C) of the source for outbreaks simulated with Scenario E, based on the first 5 (blue), 10 (red), 15 (green) observed cases. (D) Ratio of the number of individuals inaccurately targeted (IC) by the mitigation strategy for a risk threshold of 1/100,000 relative to the theoretical number of individuals at risk (%). Triangles indicate estimates for simulations in which there is no observed case infected during an occasional movement. Rectangles indicate estimates for simulations in which there is at least one observed case infected during an occasional movement. The horizontal and vertical lines indicate the true values. The third line is the bisector.

Finally, if some of the observed cases had been exposed during an occasional stay in a ward different from their home (for night release) or workplace (for day release) as in scenario E, our back-calculation method could fail to identify the actual source location. The release date estimates remained accurate (RMSE was about 9 hours for *T*) but the quality of the height and strength estimates was impaired (for estimates based on 5 cases, RMSE was 320 m for *H* and 0.97 for log_10_(*S*) versus 85 m and 0.46 respectively for the reference scenario). Indeed, for several simulations, one or more cases did not live or work within the exposed area but to encompass these cases in the estimated exposed area, the release location estimates were chosen upwind of the real location, also affecting the height and strength estimates (see Text S1). Increasing the number of observed cases did not necessarily improve the quality of estimates as it increased the probability to observe cases infected during an occasional stay in a ward different from their home or workplace. To avoid this issue, we modified the model embedded in the back-calculation; for simulations where at least one case had been infected during an occasional movement, location estimates derived from this modified model were much improved (Figure 3). Overall, with this later model, the quality of estimates improved with the number of observed cases (see Text S1) though the distance to the real source RMSE was greater when estimates were based on 10 rather than 5 observed cases.

### Comparison with other back-calculation methods

The comparison of the release date and outbreak size estimates provided by previously published methods with our results shows that performance of the three methods were similar (see Text S1).

### Implication for mitigation policies

**Figure 4:**
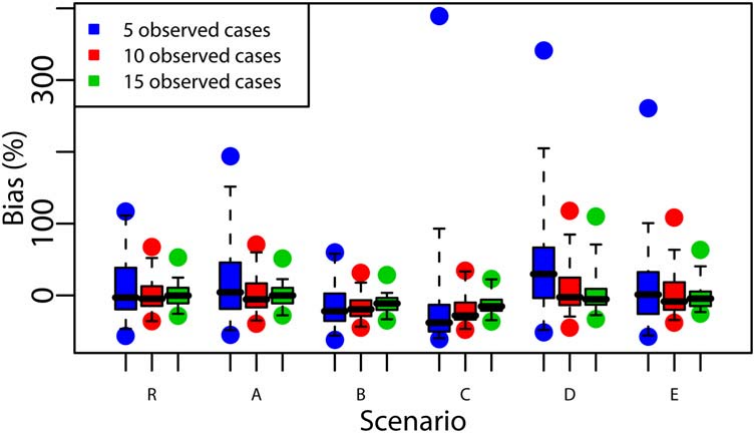
Performance of the back-calculation method to predict the outbreak size with Reference scenario (R) and scenarios A to E. Each box-plot represents the distribution (minimum, maximum, percentiles 2.5,25,50,75,97.5) of the predicted outbreak size relative bias based on the 5, 10 and 15 first cases on 40 simulated outbreaks per scenario.

Figure 4 shows that outbreak size estimates were accurate up to an order of magnitude but that relative bias for the reference scenario was up to 120% with estimates based on the first 5 cases and up to 70% with estimates based on 10 cases.

**Figure 5:**
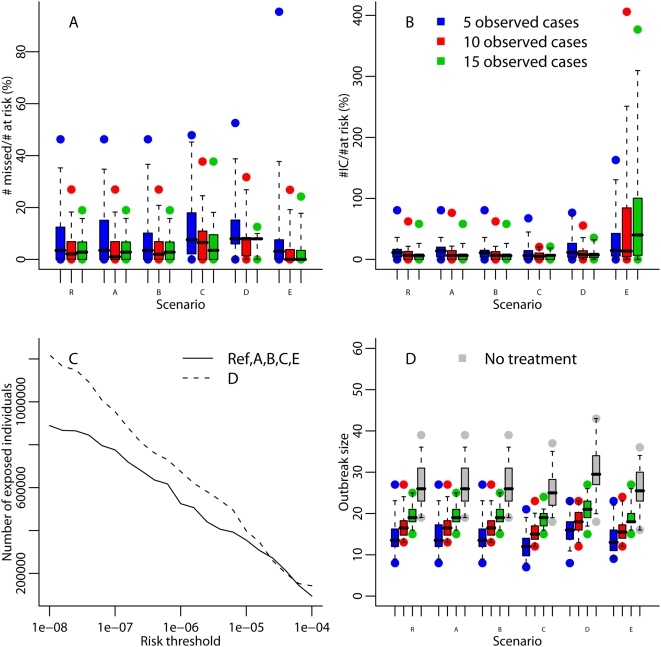
Impact of the targeting mitigation strategy with Reference scenario (R) and scenarios A to E. (A) Ratio of the number of individuals missed by the targeting mitigation strategy for a risk threshold of 1/100,000 relative to the theoretical number of individuals at risk. (B) Ratio of the number of individuals inaccurately targeted by the mitigation strategy for a risk threshold of 1/100 000 relative to the theoretical number of individuals at risk. (C) Number of individuals at risk according to the model used to generate the data. (D) Impact of administrating treatments to individuals living or working in a ward exposed to a risk of at least 1/100 ,000 inhabitants: outbreak size when there is no treatment and when prophylactic treatment compliance and efficacy is 100% prior to the onset of symptoms and administered 4 days after the first 5, 10 or 15 cases occurred. Each box-plot represents the distribution (minimum, maximum, percentiles 2.5, 25, 50, 75, 97.5) of the total number of cases.

Regarding mitigation policies, key is how many people might be missed by a risk-targeted strategy guided by the model estimates, and how many would be inaccurately considered at risk. Both of these numbers varied substantially from one simulated outbreak to another (see Figure 5). For a risk threshold of 1 case per 100,000 inhabitants and estimates based on 5 observed cases, the median proportion of at-risk individuals missed by targeting was less than 8%, for any scenario, with 3^rd^ quartiles under 20% for all scenarios (see Figure 5a). The location of those exposed wards missed by the targeting strategy and those wards inaccurately considered at risk is shown in Text S1. For any scenario other than E and estimates based on 10 or 15 observed cases, the median number of individuals inaccurately considered at risk was about 5–8% of those actually at risk (see Figure 5b) but was larger when the simulated outbreaks included local occasional movements (see scenario E, estimates based on 15 cases). Most of the wards inaccurately considered as exposed with scenario E estimates are in the west of the exposed area (see Text S1). Figure 5c shows the actual numbers at risk as a function of the risk threshold used. For Scenario E, using a model which took account of occasional movements decreased the number of individuals inaccurately considered at risk (see Figure 3d).

On average, the impact of the targeting strategy on outbreak size was greater when applied after the 5 first cases have occurred (see Figure 5d). With the reference scenario and estimates based on 5 cases, 221,000 to 642,000 individuals were treated and 1 to 21 cases were avoided (median = 12.5 cases). With the release features we used for the simulation, using a risk threshold of 1/100,000 seemed efficient: a higher threshold decreased the number of lives saved while a lower threshold did not save significantly more lives but required substantially larger numbers to be treated (see Text S1).

As shown in Text S1, on average, the strategy based on our estimates seems to be more efficient than a “ring strategy” around the 3 most affected wards (J = 3) which could require more antibiotic courses to prevent an equivalent number of cases. With Scenario E, our back-calculation method embedding the model taking into account local occasional movements seemed to be the most efficient.

## Discussion

Here we have developed and tested a back-calculation model to characterize an airborne release of anthrax spores from data on the first observed cases, meteorological conditions, population density and movement data. Our simulation study shows that this method could provide accurate results even after only a few cases of a small outbreak have been observed.

Overall, in the event of an outdoor airborne release, the source location could accurately be identified although misspecifications of the spore dispersion model (scenario D) might slightly affect the quality of the estimates. Indeed, for a given dose, the HPAC model gave a larger geographical extent of the release than the puff model (see Figure 5c) affecting source location estimates; these differences might be partly explained by differences in the dispersion parameters of the two models. Our results suggest that increasing the number of observed cases would improve the source location estimates substantially. Different spore dispersion models have been proposed [7] and could be tested in further uncertainty analyses of our back-calculation estimates; if an instantaneous exposure was still considered a reasonable assumption then the spore dispersion model component could be easily modified in our algorithm.

In the spore dispersion model we used, we set the wind direction and speed at a fixed value both in the outbreak simulations and the back-calculation algorithms. However, our method could be refined to integrate more sophisticated datasets allowing the meteorological conditions to vary with time and to be imperfectly recorded.

The source location estimate would also probably be affected by misspecifications of population movements (scenario E). Indeed, if one or more observed cases had been exposed during local (or long) distance occasional movements then the quality of estimates would be impaired. We therefore developed a modified model that allowed for exposure due to occasional movements. Including this model in the back-calculation algorithm improved the location estimates when occasional movements were included in the simulated data, although the computational time required for estimation increased markedly. Hence, the standard model could provide a first set of estimates which could then be refined using the more elaborate model with occasional movements included.

The release date estimate might be biased if the within-host dynamics, and consequently the incubation period, were misspecified (scenarios B and C): different incubation period distributions could also be tested in further uncertainty analyses. Also, the within-host model used here could be extended to deal with continuous, rather than instantaneous releases, though this would require further development of the incubation period models which have been proposed for inhalation anthrax [9],[10]. Lastly, the impact of under-reporting of cases remains to be examined (we assumed a 100% reporting rate) but is likely to only affect estimates of the overall size of release, and perhaps its timing (if under-reporting varies through time). On the contrary, a lack of specificity might bias the source location estimates. Though this remains to be evaluated, the location estimates provided by the second model we introduced might be less sensitive to false cases.

Our analysis shows that characterizing an outbreak would help to predict its final size and to assist in targeting the exposed population requiring prophylactic treatment. Although the exposed population cannot be precisely estimated (both the number of missed individuals and inaccurately targeted individuals could be substantial), treating the population estimated to be at risk using our back-calculation method could substantially reduce the number of symptomatic cases, and therefore deaths. However, our estimates of the number of cases which might be prevented represent a best case: we assumed that both compliance with treatment and its efficacy were 100% prior to the onset of symptoms. Further analysis should be carried out to take into account the impact of sub-optimal compliance and lower treatment efficacy [20]. How other parameters such as the incubation period distribution or the delay between outbreak detection and treatment would affect the efficacy of mitigation strategies and their impact remains to be explored.

A limitation of our method is the assumption of a common single source outbreak. If the outbreak was due to multiple releases, the spore dispersion component of our model could be modified to account for several sources. However, this would increase the number of parameters to estimate (four for each source) and could make the estimation based on a small number of observed cases less accurate or impossible. In addition, determining the number of sources could also be challenging. This problem might depend on the spatial separation of the sources; very widely spaced and more discrete “clusters” of cases might be quite obvious allowing their independent analysis. Some epidemiological oversight would obviously be key in such circumstances.

Our estimates could be used to parameterize models which have been developed to estimate the optimum duration of antibiotic treatments [3],[20],[21] and to evaluate various mitigation interventions following an anthrax release [15]. Other work in this latter area has shown that rapidity of intervention would be a key issue for the control of an outbreak and has proposed the use of biosensors. Better characterizing the release with the method we propose and thus estimating which areas were exposed would also help to decrease the delay in planning a targeted emergency response; it could also represent an alternative tool if biosensor data were not available.

Comparing our model with others in the literature, previous models provided estimates of the release date and the outbreak size but not the location [4],[18],[19]. Furthermore, the performance of our method was equal to that of the existing models at estimating both the release date and the outbreak size. All such back-calculation methods require knowledge about the timing of symptom onset which may not always be captured by early outbreak investigation studies depending on the systems that are in place. If hospital admission dates were available instead, the release date estimate could be biased though we have shown that the date estimate is only slightly sensitive to a 12 hours uncertainty in symptom onset dates. However, our model could also be refined to integrate a delay between symptomatic onset and hospital admission (see Hogan's presentation in http://www.galaxy.gmu.edu/QMDNS2007/). Incorporating the date of symptomatic onset and also the residence and workplace of cases into surveillance systems could shorten the delay between the occurrence of the first cases and the implementation of relevant mitigation strategies, notably by allowing the use of appropriate analytical methods, such as the one we propose here, as soon as possible. In the event of an anthrax outbreak in GB, we are anticipating having the data from detailed field epidemiological studies which should in most cases include symptoms onset dates and home/work locations (see for example the legionella outbreak investigation guidelines http://www.hpa.org.uk/web/HPAwebFile/HPAweb_C/1194947321368).

We have focused on evaluating our spatial back-calculation model for small outbreaks. In the case of a large outbreak, the rapid accumulation of cases and their locations would probably allow localization of the exposure event without the need for sophisticated methods. Nonetheless, the methods we developed here could be used for large outbreaks if statistical rigor was a key requirement for any analysis and to help with the early identification of the spatial extent of the release and the geographical targeting of antibiotic therapy. Application of this type of model to the airborne release of an agent capable of being transmitted from person-to-person (e.g. smallpox or pneumonic plague) would be feasible at the very beginning of an epidemic (before any transmission is likely to have occurred). But if secondary cases were suspected, our method would need further development to take into account the transmission process.

## Supporting Information

Text S1 SUPPLEMENTARY MATERIAL
(1.23 MB DOC)
