# Supplementary material for: Estimating the Location and Spatial Extent of a Covert Anthrax Release
Source: PLoS Comput Biol. 2009 Apr 10;5(1):e1000356. doi: 10.1371/journal.pcbi.1000356 (PMC2663800; doi:10.1371/journal.pcbi.1000356)
Supplement: Text S1 — SUPPLEMENTARY MATERIAL (1.23 MB DOC) [file pcbi.1000356.s001.doc]

**SUPPLEMENTARY MATERIAL**

TABLE OF CONTENTS

[SUPPLEMENTARY METHODS 3](#__RefHeading___Toc224457308)

[Exposure Model 3](#__RefHeading___Toc224457309)

[Figure S1: chart flows. A) Full model B) Simplified model 4](#__RefHeading___Toc224457310)

[Accounting for occasional movements 8](#__RefHeading___Toc224457311)

[Posterior Probability 8](#__RefHeading___Toc224457312)

[Estimation procedure 10](#__RefHeading___Toc224457313)

[Measuring model performance 11](#__RefHeading___Toc224457314)

[Implication for outbreak control 12](#__RefHeading___Toc224457315)

[SUPPLEMENTARY TABLES 14](#__RefHeading___Toc224457316)

[Table S1: Performance of the back-calculation method in estimating the height of the release. 14](#__RefHeading___Toc224457317)

[Table S2: Performance of the back-calculation method in estimating the strength of the release. 15](#__RefHeading___Toc224457318)

[Table S3: Performance of the back-calculation method in estimating the time of the release. 16](#__RefHeading___Toc224457319)

[Table S4: Performance of the back-calculation method in estimating the release location. 17](#__RefHeading___Toc224457320)

[Table S5: Performance of the back-calculation method in predicting the observed outbreak size. 18](#__RefHeading___Toc224457321)

[Table S6: Performance of the back-calculation method using the modified model (integrating occasional movements) to characterize the release and estimate the observed outbreak size. 19](#__RefHeading___Toc224457322)

[SUPPLEMENTARY FIGURES 20](#__RefHeading___Toc224457323)

[Figure S2: Population size of the 10 515 wards based on the 1991 census data 20](#__RefHeading___Toc224457324)

[Figure S3: Localization of the workplaces (blue dots) and home locations (red dots) of cases in 40 simulated outbreaks for each scenario. 21](#__RefHeading___Toc224457325)

[Figure S4: Distribution of the delay between the release and the symptoms onset dates for cases in 40 simulated outbreaks (a) and distribution of the 40 outbreak sizes (b) for each scenario. 21](#__RefHeading___Toc224457326)

[Figure S5: Histograms of the release date estimates for the 40 simulated outbreaks with Reference scenario (Ref.) and scenarios A to E. 22](#__RefHeading___Toc224457327)

[Figure S6: Example of posterior distribution of the release height (a), strength (b), date (c) and location (d) for one outbreak generated with the reference scenario and estimates based on the first 5, 10 or 15 observed cases. 23](#__RefHeading___Toc224457328)

[Figure S7: Point estimates (calculated posterior distribution median) of release (a) height and (b) strength from 40 simulated datasets for the Reference scenario (R) and scenarios A to E. 23](#__RefHeading___Toc224457329)

[Figure S8: A simulated outbreak with scenario E and release feature estimates based on the standard model (M1, second line) and based on the model allowing for occasional movements during the day (M2, third line). 24](#__RefHeading___Toc224457330)

[Figure S9: Performance of the back-calculation method when including the model allowing for occasional movements during the day to characterize releases simulated with scenario E 25](#__RefHeading___Toc224457331)

[Figure S10: Performance of spatio-temporal back-calculation method compared with other published methods when 5 (a and b), 10 (c and d) or 15 (e and f) cases are observed. 26](#__RefHeading___Toc224457332)

[Figure S11: Number of simulations for which people living or working in a given ward would be missed by targeted controls (red dots) or inaccurately targeted (green dots). 27](#__RefHeading___Toc224457333)

[Figure S12: Impact of administering treatment to individuals living or working in a ward exposed to a given risk (reference scenario): outbreak size when there is no treatment (grey box-plots) and when a 100% effective prophylactic treatment is administered 4 days after the first 5 (a), 10 (b) or 15 (c) cases occurred. 28](#__RefHeading___Toc224457334)

[Figure S13: Comparison of the outbreak size when the mitigation strategy based on our estimates was applied and when a ring strategy around the 3 most infected wards (J=3) was applied. 29](#__RefHeading___Toc224457335)

[REFERENCES 30](#__RefHeading___Toc224457336)

# SUPPLEMENTARY METHODS

## Exposure Model

***The dispersion of anthrax spores in the air: a puff model***

The average ground level spore concentration at time *t*, *x* meters downwind from the source and *y* meters crosswind from the source is given by the following puff model [1]:

$$\left\langle c\left(x,y,0,t\right)\right\rangle=\frac{2S}{\left(2\pi\right)^{3/2}\sigma_{x}\sigma_{y}\sigma_{z}}e^{-\frac{\left(x-\overline{u}\left(t-T\right)\right)^{2}}{2\sigma_{x}^{2}}-\frac{y^{2}}{2\sigma_{y}^{2}}-\frac{H^{2}}{2\sigma_{z}^{2}}-\gamma\left(t-T\right)}$$

where *S* is the number of spores released (also referred to as “strength”) from height *H*, at time *T*, $\bar{u}$is the average wind velocity, *γ* is the anthrax spores decay rate and dispersion parameters $\sigma_{x}\text{, }\sigma_{y}\text{, }\sigma_{z}$ are given by the Briggs formulations, also used by Wein et al and Stuart and Wilkening [2,3].

***The within-host dynamics probabilistic model***

Once an anthrax spore is inhaled, we assume it can either germinate or be cleared from the lungs, as represented in Figure S1.A [4]. We assume a constant hazard for both processes, with the hazard rate of germination being *λ* and the hazard rate of clearance being *θ*. Following germination, spores have a growth rate *r*.

Figure S1: chart flows. A) Full model B) Simplified model

We can represent the within-host dynamics as a birth-death process with migration as represented in Figure S1.A [5]. Let *N*(*t*) and *M*(*t*) be the number of non germinated and germinated spores. The joint distribution of *N(t)* and *M(t)* is described by the following system of ordinary differential equations, where *q_n m_ (t)=*P(*N*(*t*) =*n, M*(*t*) *=m*).

$$\begin{matrix} \left\{\begin{matrix} \text{If }n\geq1,m\geq1 \\ q_{n,m}^{'}\left(t\right)=r\left(m-1\right)q_{n,m-1}\left(t\right)+\lambda\left(n+1\right)q_{n+1,m-1}\left(t\right)+\theta\left(n+1\right)q_{n+1,m}\left(t\right) \\ +d\left(t\right)q_{n-1,m}\left(t\right)-(rm+\alpha n+d\left(t\right))q_{n,m}\left(t\right) \\ \text{ } \\ \text{If }n=0,m\geq1 \\ q_{0,m}^{'}\left(t\right)=r\left(m-1\right)q_{0,m-1}\left(t\right)+\lambda q_{1,m-1}\left(t\right)+\theta q_{1,m}\left(t\right)-(rm+d\left(t\right))q_{0,m}\left(t\right) \\ \\ \text{If }n\geq1,m=0 \\ q_{n,0}^{'}\left(t\right)=\theta\left(n+1\right)q_{n+1,0}\left(t\right)+d\left(t\right)q_{n-1,0}\left(t\right)-(\alpha n+d\left(t\right))q_{n,0}\left(t\right) \\ q_{0,0}^{'}\left(t\right)=\theta q_{1,0}\left(t\right)-d\left(t\right)q_{0,0}\left(t\right) \\ \end{matrix}\right. \\ \text{with }\theta\text{, }\lambda\text{, }r\text{ defined as above, }\alpha=\theta+\lambda\text{ } \\ \text{and }d(t)=b\left\langle c(t)\right\rangle\text{ with }\left\langle c(t)\right\rangle\text{ given by the puff model described in equation (1.1)} \\ \text{and }b\text{ the breathing rate.} \end{matrix}$$

However, it was not possible to infer a simple analytical solution for *q_n,m_*. For this reason, we have considered the within-host dynamics of anthrax spores as the superposition of a Poisson process due to the germination process (see Figure S1.B1) and a Negative Binomial Process [5] due to the growth process (see Figure S1.B2), the latter starting after the germination of the first spore. Given the very different timescales of the two processes, we have considered that once the growth process has started, the germination process can be neglected. Hence, it can be shown that *F*_1_, the cumulative probability that one spore germinates is

$$F_{1}(t)=1-P(M(t)=0)=1-\exp\left(-\frac{\lambda}{\alpha}\left(A\left(t,\gamma\right)-e^{-\alpha\left(t-T\right)}A\left(t,\gamma-\alpha\right)\right)\right)$$

where *A*(*t*,*γ*) is defined as:

$$\begin{matrix} A\left(t,\gamma\right)=\frac{bS}{\pi\bar{u}}\sum_{i=0}^{I} \left(\frac{e^{-\frac{y_{i}^{2}}{2\sigma_{x_{i}}^{2}}-\frac{H^{2}}{2\sigma_{z_{i}}^{2}}+\frac{\gamma^{2}\sigma_{x_{i}}^{2}}{2\bar{u}^{2}}-\frac{\gamma x_{i}}{\bar{u}}}}{\sigma_{x_{i}}\sigma_{z_{i}}}\right. \\ \left.\times\left[\Phi\left(\frac{\bar{u}\left(\chi_{i+1}-T\right)}{\sigma_{x_{i}}}-\left(\frac{x_{i}}{\sigma_{x_{i}}}-\frac{\sigma_{x_{i}}\gamma}{\bar{u}}\right)\right)-\Phi\left(\frac{\bar{u}\left(\chi_{i}-T\right)}{\sigma_{x_{i}}}-\left(\frac{x_{i}}{\sigma_{x_{i}}}-\frac{\sigma_{x_{i}}\gamma}{\bar{u}}\right)\right)\right]\right) \end{matrix}$$

with

- Φ denoting the standard normal cumulative distribution
- {*χ*_i_}i=1,…I being the times at which an individual move from one ward to another between 0 and *χ*_I+1_=t
- (*x_i_*,*y_i_*,0) the location of the individual between *χ_i_* and *χ_i_*_+1_.

Commuting times {*χ*_i_} depend on the way population movements are modeled (see Spatio-temporal distribution of disease incidence).

It can also be shown that *G_k_*, the cumulative distribution function of the time from germination of the first spore to the time at which the number of spores reaches a given threshold k is

$$G_{k}\left(t\right)=1-\sum_{m=1}^{k-1} e^{-rt}\left(1-e^{-rt}\right)^{m-1}=\left(1-e^{-rt}\right)^{k-1}$$

To account for the delay between the germination of the first spore and the occurrence of symptoms, we considered that symptoms would occur after the number of germinated spores has reached a given threshold *k*, at time *T_D_*. The cumulative attack probability function is given by$F_{k}(t)=P(T_{D}\leq t)=P(M(t)\geq k)=1-P(M(t)<k)$.

It can be shown that

$$F_{k}(t)=\int_{0}^{t} G_{k}\left(t-u\right)f_{1}(u)du=\int_{0}^{t} g_{k}\left(t-u\right)F_{1}(u)du$$

where $F_{1}^{'}\left(t\right)=f_{1}\left(t\right)$and $G_{_{k}}^{'}\left(t\right)=g_{k}\left(t\right)$

Other distributions have been proposed for the time lag from germination to symptoms such as an exponential distribution [4] or a Log-Normal distribution [6]. To simplify further, we have considered the growth process as a deterministic process leading to the cumulative attack probability function:

$$F_{k}(t)=\left\{\begin{matrix} F_{1}\left(t-\frac{\log(k)}{r}\right)\text{ where }t\text{>}\frac{\log(k)}{r} \\ 0\text{ elsewhere} \end{matrix}\right.$$

***Spatio-temporal distribution of disease incidence***

To determine the spatio-temporal dynamics of the outbreak, we integrate the spore-dispersion model, the within-host dynamics model and population density and movements from the 1991 GB census for the 10,515 wards provided by the Office for National Statistics. We considered that each individual stays at his/her workplace from 9 am to 7 pm and his/her residence from 7 pm to 9 am. Hence, in equation , {*χ*_i_}_i=0,…I_ will be set at 9 am and 7 pm each day between 0 and t. We used this formulation to simulate the data but the model embedded in the back-calculation method assumed an instantaneous exposure at time *T* with an absorbed dose given by $\mathop{\lim A\left(t,\gamma\right)}\limits_{t\to\infty}$ (taking into account the location at *T* only) giving:

$$F_{1}(t)=1-\exp\left(-\frac{\lambda}{\alpha}Dose\left(1-e^{-\alpha\left(t-T\right)}\right)\right)$$

which is the equivalent to the cumulative probability that one spore germinates derived by Brookmeyer et al [4].

## Accounting for occasional movements

We also considered a modified version of the model described above in order to take into account occasional movements during the day. We considered that people would have a probability ς to travel away from their working place and as a consequence to be exposed in another ward. If we denote *F_ki_* the cumulative attack probability function in ward i given by equation (1.7), then, the cumulative attack probability function in the new model will be:

$$\begin{matrix} H(t)=(1-\varsigma)F_{ki}(t)+\varsigma\sum_{j\in J_{i}} \omega_{ij}F_{kj}(t) \\ \text{with }\omega_{ij}=\frac{N_{j}}{\sum_{l\in J_{i}} N_{l}}\text{ and }J_{i}=\left\{j|Dist(W_{i},W_{j})<D_{m},j\neq i\right\} \\ W_{i}\text{ denoting wards, }N_{i}\text{ denoting the number of individuals in ward }W_{i}\text{ during the day and } \\ D_{m}\text{ the maximum distance between the usual workplace and the occasional destination.} \end{matrix}$$

We considered that the cumulative attack probability function would remain unchanged for a night time release.

## Posterior Probability

Let’s assume that we observe a censored realization of the model described above, up to censoring time τ. For each individual *i* in the GB population (denoted *I*), *t_i_* represents the onset date of symptoms (with the convention that *t_i_*=∞ for non infected individuals), $w_{i}^{H}$ the ward of residence and $w_{i}^{W}$ the workplace ward of the individual. The collected data are then $Y=\left\{t_{i},w_{i}^{H},w_{i}^{W}\right\}_{i\in I}$.

In a Bayesian setting, the posterior distribution of the parameters is

$$P\left(\Theta|\left\{t_{i},w_{i}^{H},w_{i}^{W}\right\}_{i\in I}\right)\propto P\left(\left\{t_{i}\right\}_{i\in I}|\left\{\Theta,\left\{w_{i}^{H},w_{i}^{W}\right\}_{i\in I}\right\}\right)P\left(\Theta\right)$$

where $\Theta=\left(T,H,\log_{10}(S),W\right)$ is the set of parameters. The first term on the right hand side of equation is the likelihood; the second term is the prior density. Prior uniform distributions are specified for the date (*T*∈[-3000+*t*_min_, *t*_min_], with $t_{\min}=\min\left\{t_{i}\right\}_{i\in I}$), the height (*H*∈[0,3000]) and the strength (log(*S*) ∈[0,16]; on a log_10_ scale).

If *J* is the set of cases who have been observed by the censoring date *τ* , we have

$$P\left(\left\{t_{i}\right\}_{i\in I}|\left\{\Theta,\left\{w_{i}^{H},w_{i}^{W}\right\}_{i\in I}\right\}\right)=\prod_{j\in J} \rho\left(t_{j}\left|t_{j}<\tau,\Theta,\left\{w_{j}^{H},w_{j}^{W}\right\}\right.\right)\prod_{j\notin J} P\left(t_{j}>\tau\left|\Theta,\left\{w_{j}^{H},w_{j}^{W}\right\}\right.\right)\text{ }$$

Here *ρ*( *t *.)dt is the probability of having onset time in [t;t+dt] given that symptom onset occurs before time *τ* :

$$\rho\left(t|.\right)=\frac{p_{j}f_{j}\left(t\right)}{F_{j}\left(\tau\right)}$$

where *F_j_*(*t*) is the cumulative probability of developing symptoms by time *t* for individual *j* (given by the model described above; to simplify the notation, we have dropped the subscript for the threshold *k* from *F*()),$p_{j}=\lim_{t\to\infty} F_{j}\left(t\right)$is the risk of ever developing the disease for individual *j* and $f_{j}=dF_{j}/dt$. The probability of not being observed as a case (*i.e* latent or uninfected) by time *τ* is $P\left(t_{j}>\tau\left|\Theta,\left\{w_{j}^{H},w_{j}^{W}\right\}\right.\right)=1-F_{j}\left(\tau\right)$.

## Estimation procedure

We initially tried to explore the posterior distribution presented in equation with a standard Markov Chain Monte Carlo (MCMC) algorithm. The three parameters {*T*,*H*,*S*} were highly correlated to source location *W*, which prevented reaching satisfying mixing properties for the chain.

Following [7-9], we therefore relied on the profile likelihood of the 3-dimension parameter space $\left\{T,H,S\right\}$:

$$lp\left(\left\{T,H,S\right\}|Y\right)=L\left(\left\{T,H,S,\hat{W}\left(T,H,S\right)\right\}|Y\right)$$

where *L*(.) is the likelihood function presented in equation and $\hat{W}\left(T,H,S\right)$maximizes $L\left(\left\{T,H,S,W\right\}|Y\right)$with respect to *W*.

Parameter space $\left\{T,H,S\right\}$was explored with a standard Metropolis-Hastings algorithm. At each iteration, the source location $\hat{W}$maximizing the likelihood was also recorded.

In order to decrease the computational time required for the determination of the source location, we preselected “possible sources” and “possible exposed wards” before running the MCMC algorithm. We selected as “possible source” all wards in which a release at height *H*=0 of 10^16^ spores would lead to a risk of developing symptoms of 10^-6^ in either the workplace ward or the residence ward of all observed cases. We preselect as “possible exposed wards” those for which a release of 10^16^ spores at height 0 in any of the possible sources would lead to a risk of developing symptoms greater than 10^-6^ or, wards connected with such wards via commuter movements. This pre-selection decreases the number of possible sources and exposed wards. As a test that this pre-selection did not affect the results, we ran the back-calculation method with a 10^-9^ risk threshold on one simulated outbreak (first five cases) and obtained similar results as with a 10^-6^ risk threshold.

For scenario E (see main text), the selection of “possible sources” was modified. Instead of selecting sources as source locations for which all cases would be exposed to a risk of 10^-6^, we selected source locations for which at least one observed case would be exposed to this risk.

## Measuring model performance

As described in the main text, we first simulated 40 outbreaks with the model and we measured the performance of the back-calculation method based on 5, 10 and 15 observed cases. For each simulated outbreak, the outputs are the posterior distributions of the date (*T*), the strength (log_10_*S*), the height (*H*) and the location (*W*) of the release.

The performance of the method was measured by the root mean square error (RMSE) of the estimates. For each scenario and each parameter *X*, we define the root mean square error (RMSE) as:

$$RMSE=\sqrt{\frac{\sum_{i=1}^{40} \left(\hat{X}_{i}-X\right)^{2}}{40}}\text{ }$$

where $\hat{X}_{i}$ is the estimate for scenario *i* and *X* is the real parameter value. In addition, we also measured how well the estimated outbreak size matched the expected outbreak size. The posterior distribution of the estimated outbreak size was computed using the posterior distribution of the four estimated parameters. For each set of parameters, assuming an instantaneous dose, we computed the attack rate in each ward given by the limit of equation (1.8), multiplied by the number of individuals exposed at the release date and then summed over all wards. We have computed the root mean square relative error as follows:

$$RMSE1=\sqrt{\frac{\sum_{i=1}^{40} \left(\frac{\hat{Y}_{i}-Y_{\exp}}{Y_{\exp}}\right)^{2}}{40}}\text{ }$$

where $\hat{Y}_{i}$is the estimated outbreak size for scenario *i* and *Y*_exp_ is the expected outbreak size computed with the real parameter values.

We have also computed the coverage defined as the probability that the real (or expected) value falls in the (2.5^th^,97.5^th^) percentiles interval of the estimated value. Estimates could be more accurate according to the RMSE although having a smaller coverage due to narrower posterior distributions.

## Implication for outbreak control

We have also measured how well our method would predict the observed outbreak size by measuring how the estimated outbreak size conditioned by the censoring time *τ* fit the observed outbreak size. For an individual exposed to an instantaneous dose at the estimated release time and not showing symptoms at *τ*, the probability to show symptoms between *τ* and +∞ was computed as $\frac{\lim_{t\to\infty} F_{1}(t)-F_{1}(\tau)}{1-F_{1}(\tau)}$.

We have defined RMSE2 as follows:

$$RMSE2=\sqrt{\frac{\sum_{i=1}^{40} \left(\frac{\hat{Y}_{i}-Y_{i}}{Y_{i}}\right)^{2}}{40}}\text{ }$$

Where $\hat{Y}_{i}$is the estimated outbreak size for scenario *i* based on reported cases up to time *τ* and *Y_i_* is the final observed outbreak size of scenario *i*.

# SUPPLEMENTARY TABLES

Table S1: Performance of the back-calculation method in estimating the height of the release.

The posterior distribution median was used for point estimates. RMSE and absolute bias given in meters.

| **Scenario** | **Number of observed cases** | **Coverage**  **(%)** | **RMSE** | **Absolute bias range** |
| --- | --- | --- | --- | --- |
| Reference | 5 | 100 | 86 | 8-279 |
|  | 10 | 95 | 73 | 0-237 |
|  | 15 | 82.5 | 64 | 2-177 |
| A | 5 | 100 | 87 | 3-290 |
|  | 10 | 95 | 73 | 2-233 |
|  | 15 | 85 | 64 | 1-181 |
| B | 5 | 100 | 85 | 3-269 |
|  | 10 | 95 | 73 | 2-236 |
|  | 15 | 85 | 64 | 2-182 |
| C | 5 | 97.5 | 98 | 5-248 |
|  | 10 | 95 | 74 | 3-187 |
|  | 15 | 97.5 | 57 | 1-139 |
| D | 5 | 90 | 181 | 1-664 |
|  | 10 | 77.5 | 128 | 11-410 |
|  | 15 | 60 | 105 | 2-259 |
| E | 5 | 80 | 322 | 6-1291 |
|  | 10 | 65 | 359 | 2-1318 |
|  | 15 | 50 | 508 | 3-2157 |

Table S2: Performance of the back-calculation method in estimating the strength of the release.

The posterior distribution median was used for point estimates.

| **Scenario** | **Number of observed cases** | **Coverage**  **(%)** | **RMSE** | **Absolute bias range** |
| --- | --- | --- | --- | --- |
| Reference | 5 | 95 | 0.46 | 0.04-1.22 |
|  | 10 | 90 | 0.33 | 0.00-0.82 |
|  | 15 | 87.5 | 0.27 | 0.01-0.78 |
| A | 5 | 90 | 0.48 | 0.00-1.24 |
|  | 10 | 87.5 | 0.34 | 0.00-0.91 |
|  | 15 | 87.5 | 0.27 | 0.00-0.78 |
| B | 5 | 92.5 | 0.45 | 0.03-1.18 |
|  | 10 | 87.5 | 0.33 | 0.00-0.83 |
|  | 15 | 87.5 | 0.28 | 0.00-0.81 |
| C | 5 | 92.5 | 0.39 | 0.02-0.85 |
|  | 10 | 95 | 0.25 | 0.03-0.87 |
|  | 15 | 97.5 | 0.21 | 0.00-0.65 |
| D | 5 | 70 | 0.84 | 0.02-2.02 |
|  | 10 | 67.5 | 0.62 | 0.05-1.54 |
|  | 15 | 57.5 | 0.52 | 0.10-1.10 |
| E | 5 | 82.5 | 0.97 | 0.00-3.42 |
|  | 10 | 72.5 | 1.05 | 0.00-3.81 |
|  | 15 | 52.5 | 1.38 | 0.01-5.31 |

Table S3: Performance of the back-calculation method in estimating the time of the release.

The posterior distribution was discretized with day/night classes (9AM-7PM/7PM-9AM) and the centre of the modal class was used as the point estimate. RMSE and absolute bias given in hours.

| **Scenario** | **Number of observed cases** | **RMSE** | **Absolute bias range** |
| --- | --- | --- | --- |
| Reference | 5 | 12 | 5-29 |
|  | 10 | 11 | 5-29 |
|  | 15 | 12 | 5-29 |
| A | 5 | 12 | 5-29 |
|  | 10 | 13 | 5-29 |
|  | 15 | 13 | 5-29 |
| B | 5 | 65 | 43-79 |
|  | 10 | 64 | 43-67 |
|  | 15 | 64 | 43-67 |
| C | 5 | 24 | 5-43 |
|  | 10 | 24 | 5-43 |
|  | 15 | 24 | 5-43 |
| D | 5 | 9 | 5-29 |
|  | 10 | 7 | 5-29 |
|  | 15 | 7 | 5-29 |
| E | 5 | 9 | 5-29 |
|  | 10 | 8 | 5-29 |
|  | 15 | 8 | 5-29 |

Table S4: Performance of the back-calculation method in estimating the release location.

The posterior distribution peaks were used as point estimates. The bias is quantified by the distance in meters between the estimated source and the real source.

| **Scenario** | **Number of observed cases** | **RMSE** | **Absolute bias range** |
| --- | --- | --- | --- |
| Reference | 5 | 2020 | 0-7410 |
|  | 10 | 880 | 0-3770 |
|  | 15 | 650 | 0-3640 |
| A | 5 | 2160 | 0-7410 |
|  | 10 | 880 | 0-3770 |
|  | 15 | 650 | 0-3640 |
| B | 5 | 2050 | 0-7410 |
|  | 10 | 880 | 0-3770 |
|  | 15 | 650 | 0-3640 |
| C | 5 | 1350 | 0-4360 |
|  | 10 | 640 | 0-2120 |
|  | 15 | 520 | 0-2120 |
| D | 5 | 4550 | 0-18220 |
|  | 10 | 1220 | 0-3640 |
|  | 15 | 640 | 0-3640 |
| E | 5 | 14810 | 0-68560 |
|  | 10 | 13840 | 0-57350 |
|  | 15 | 22930 | 0-117470 |

Table S5: Performance of the back-calculation method in predicting the observed outbreak size.

The outbreak size was estimated by conditioning by the censoring date τ. The posterior distribution medians were used as point estimates and was compared with observed data.

| **Scenario** | **Number of observed cases** | **Coverage**  **(%)** | **RMSE2**  **(%)** | **Range of the absolute relative error (%)** |
| --- | --- | --- | --- | --- |
| Reference | 5 | 95 | 43 | 1-117 |
|  | 10 | 85 | 23 | 1-67 |
|  | 15 | 80 | 16 | 0-53 |
| A | 5 | 85 | 64 | 0-194 |
|  | 10 | 85 | 25 | 1-71 |
|  | 15 | 80 | 16 | 0-52 |
| B | 5 | 87.5 | 33 | 1-62 |
|  | 10 | 67.5 | 24 | 1-45 |
|  | 15 | 60 | 17 | 1-35 |
| C | 5 | 55 | 75 | 3-389 |
|  | 10 | 45 | 30 | 2-48 |
|  | 15 | 42.5 | 19 | 1-36 |
| D | 5 | 82.5 | 88 | 1-341 |
|  | 10 | 82.5 | 35 | 1-118 |
|  | 15 | 72.5 | 27 | 1-110 |
| E | 5 | 82.5 | 57 | 1-261 |
|  | 10 | 82.5 | 30 | 2-108 |
|  | 15 | 62.5 | 18 | 1-63 |

Table S6: Performance of the back-calculation method using the modified model (integrating occasional movements) to characterize the release and estimate the observed outbreak size.

Outbreaks were simulated with Scenario E and estimates were based on the first 5, 10, or 15 observed cases.

| **Parameter** | **Unit** | **# observed cases** | **Coverage** | **RMSE** | **Absolute bias range** |
| --- | --- | --- | --- | --- | --- |
| H | Meters | 5  10  15 | 97.5  92.5  95 | 159  119  70 | 4-554  4-400  1-225 |
| Log_10_(S) |  | 5  10  15 | 92.5  95  90 | 0.60  0.41  0.25 | 0-2.05  0.01-1.20  0.01-0.83 |
| T | Hours | 5  10  15 | - | 9  8  8 | 5-29  5-29  5-29 |
| Dist(W,W_0_) | Kms | 5  10  15 | - | 2.28  3.40  1.40 | 0-9.9  0-18.2  4.29 |
| Outbreak size | % | 5  10  15 | 90  92.5  92.5 | 88* (RMSE1)  51  35 | 3-400*  1-186  2-118 |
| Outbreak size conditioning by the censoring date τ | % | 5  10  15 | 85  82.5  82.5 | 56* (RMSE2)  30  22 | 1-253*  2-108  0-50 |

* Relative bias in % (relative to the expected outbreak size for outbreak size predictions (RMSE1) and relative to the observed outbreak size for the size conditioning by the censoring date τ (RMSE2))

# SUPPLEMENTARY FIGURES

Figure S2: Population size of the 10 515 wards based on the 1991 census data


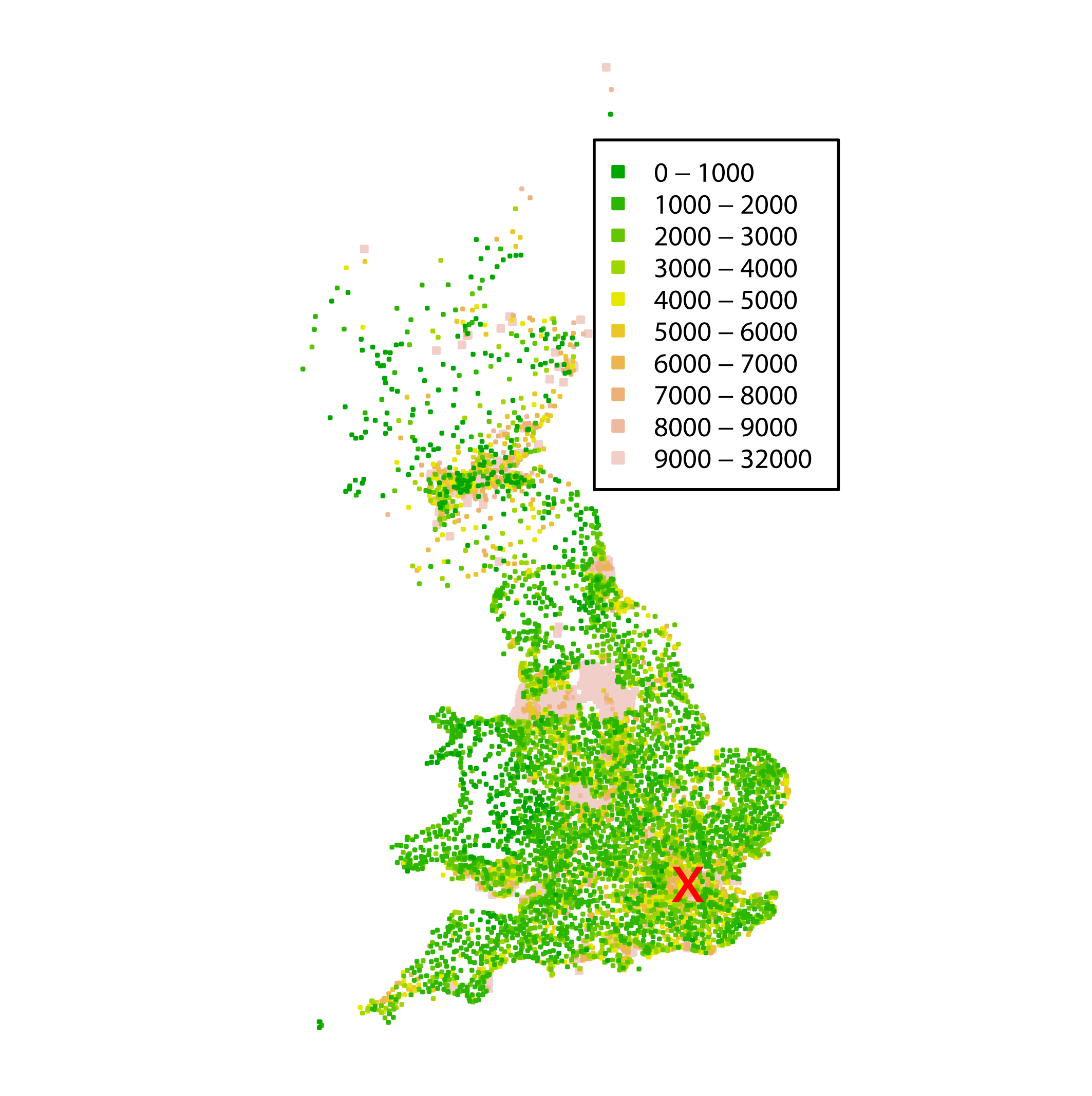


**Simulated outbreaks**

For the reference scenario and for scenarios C, D, E described in the main text, we simulated 40 outbreaks. Scenarios A and B were based on the same datasets as the reference scenario. About 30% of the cases had a workplace different from their ward of residence (see Figure S3). Using all 40 simulations, the median delay between the release and symptoms was 8.2 days (CI95%=2.2-35.1) for the reference scenario, 9.6 days (CI95%=1.5-35.1) for scenario C, 8.1 days (CI95%=2.1-33.7) for scenario D, 8.2 days (CI95%=2.2-37.1) for scenario E (see Figure S4a). The average outbreak size was 27.0 (range=19-39) for the reference scenario, 25.4 (18-37) for scenario C, 30.7 (18-43) for scenario D and 26.1 (16-36) for scenario E (see Figure S4b).

Figure S3: Localization of the workplaces (blue dots) and home locations (red dots) of cases in 40 simulated outbreaks for each scenario.


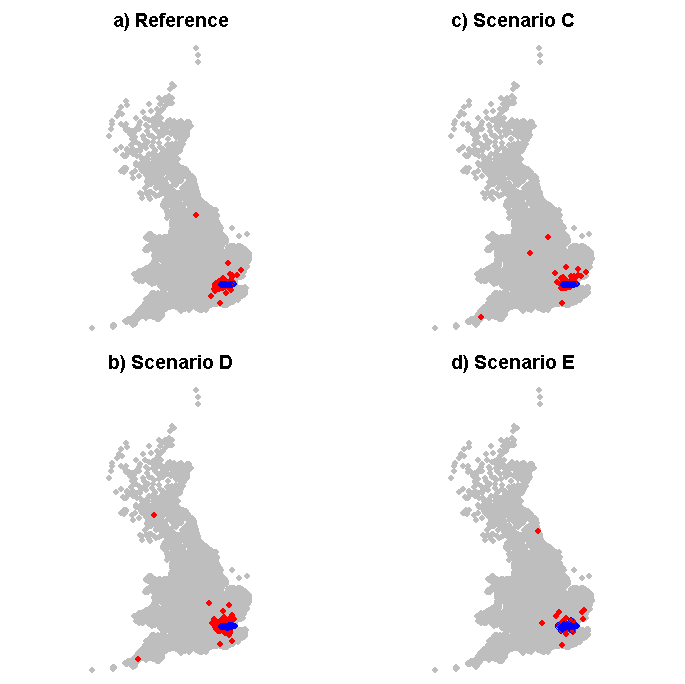


Figure S4: Distribution of the delay between the release and the symptoms onset dates for cases in 40 simulated outbreaks (a) and distribution of the 40 outbreak sizes (b) for each scenario.


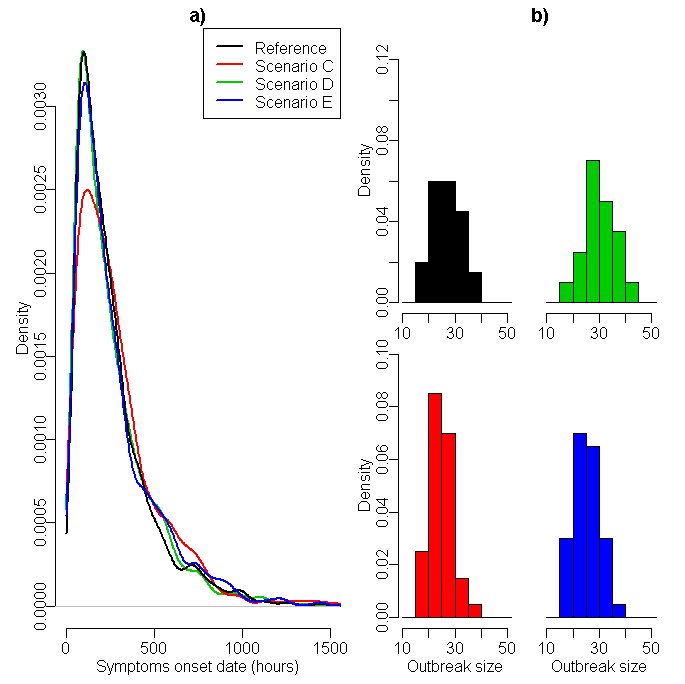


Figure S5: Histograms of the release date estimates for the 40 simulated outbreaks with Reference scenario (Ref.) and scenarios A to E.

**Left column: median as point estimates, right column: middle time of the mode class as point estimates.**


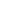


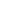

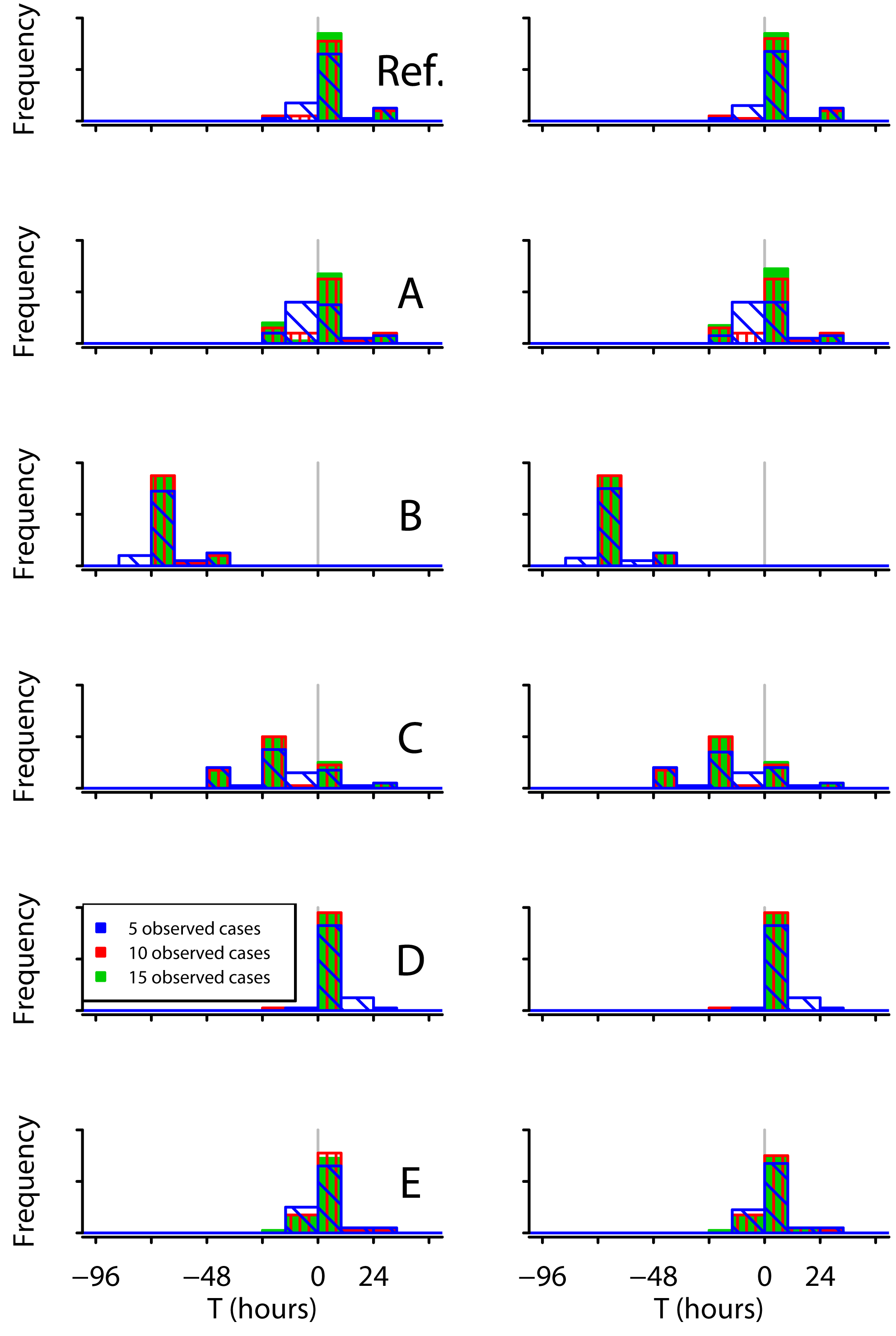


Figure S6: Example of posterior distribution of the release height (a), strength (b), date (c) and location (d) for one outbreak generated with the reference scenario and estimates based on the first 5, 10 or 15 observed cases.


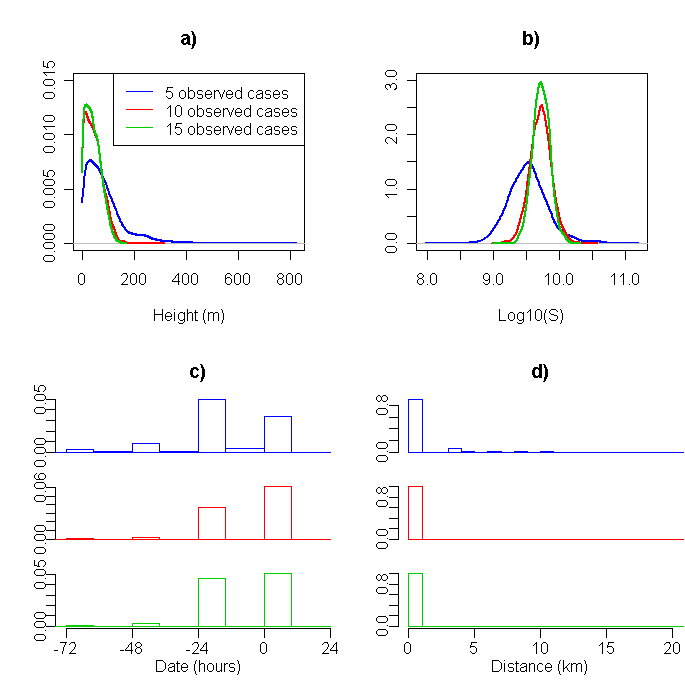


Figure S7: Point estimates (calculated posterior distribution median) of release (a) height and (b) strength from 40 simulated datasets for the Reference scenario (R) and scenarios A to E.

**Each box-plot represents the distribution (minimum, percentiles 2.5, 25, 50, 75,97.5, maximum).**


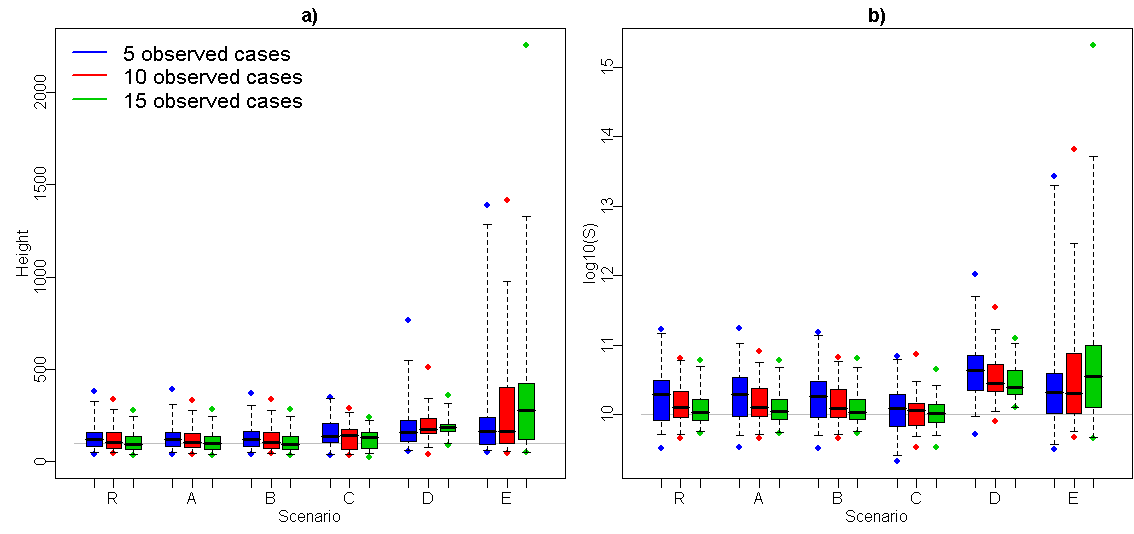


Figure S8: A simulated outbreak with scenario E and release feature estimates based on the standard model (M1, second line) and based on the model allowing for occasional movements during the day (M2, third line).

**With M1, when 5 cases are analyzed, the occasional stay of one case in his ward of residency during the day leads to the most likely estimated timing of the release being at night. When 5 more cases were observed (10 cases in total), two new cases living far from the exposed area but working in the exposed area lead to the release being estimated as occurring during the daytime and the estimated release location being slightly upstream compared to the real one. Finally, when 15 cases were analyzed, one case corresponded to an individual who worked far from the exposed area but was exposed in his residency ward where he occasionally remained during the day. As a consequence the algorithm estimated that the release took place during the night and the estimated source location was substantially upwind of the real location in order to encompass the residency wards of all observed cases.**

#
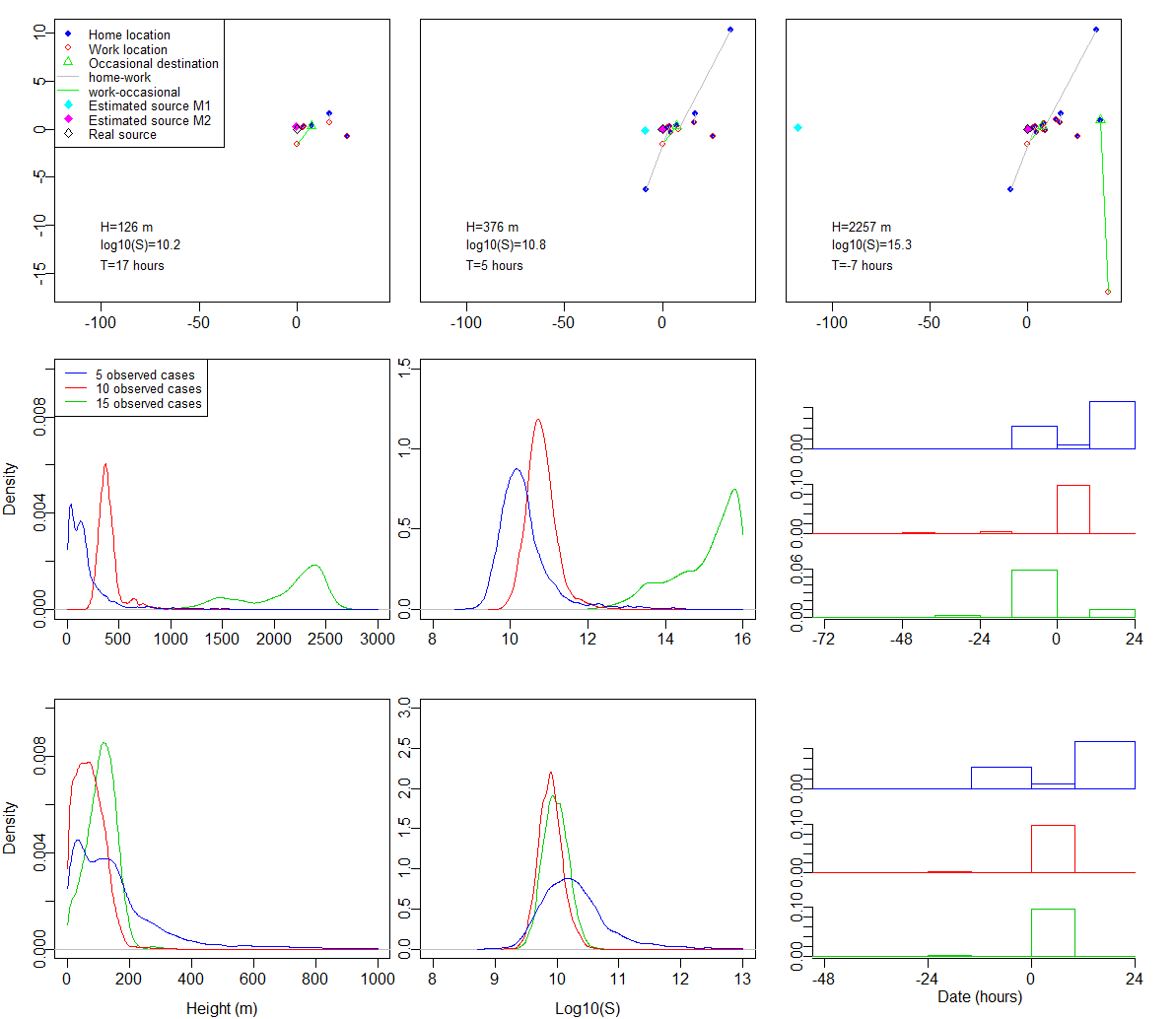


Figure S9: Performance of the back-calculation method when including the model allowing for occasional movements during the day to characterize releases simulated with scenario E

**a) Height point estimates, b) Release strength point estimates, c) Bias of the outbreak size point prediction (conditioning by the censoring date τ), d) Distance of the estimated source to the real source, e) Release date point estimates**


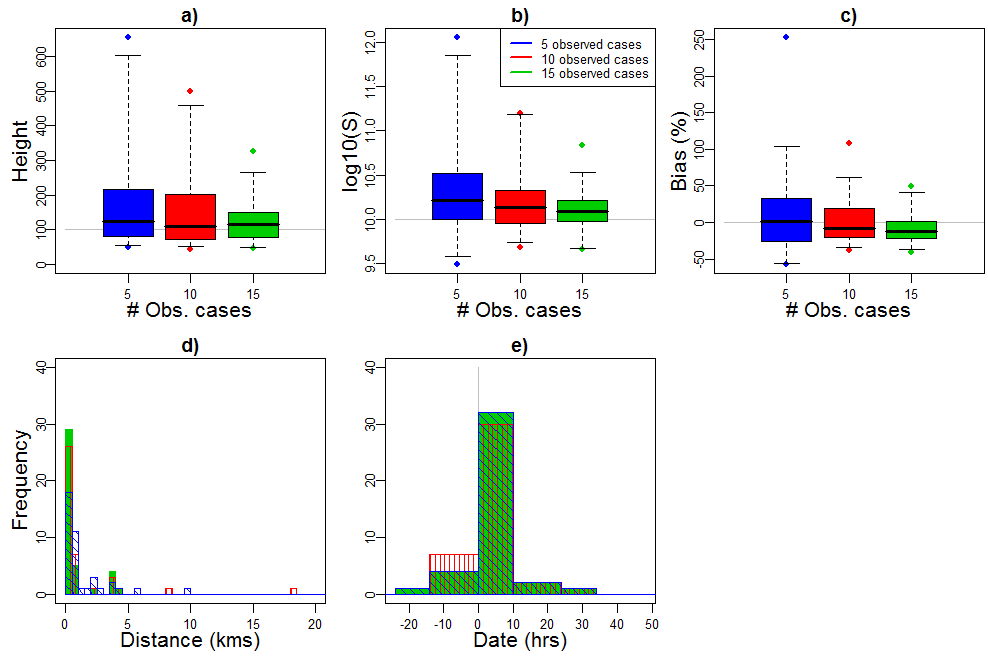


Figure S10: Performance of spatio-temporal back-calculation method compared with other published methods when 5 (a and b), 10 (c and d) or 15 (e and f) cases are observed.

**a, c and e represent the release date estimates and b, d and f represent the relative bias of the outbreak size estimates. The figures under each box-plot represent the relative root mean square error. Each box-plot represents the distribution (minimum, maximum, percentiles 2.5, 25, 50, 75, 97.5) of the total number of cases.**


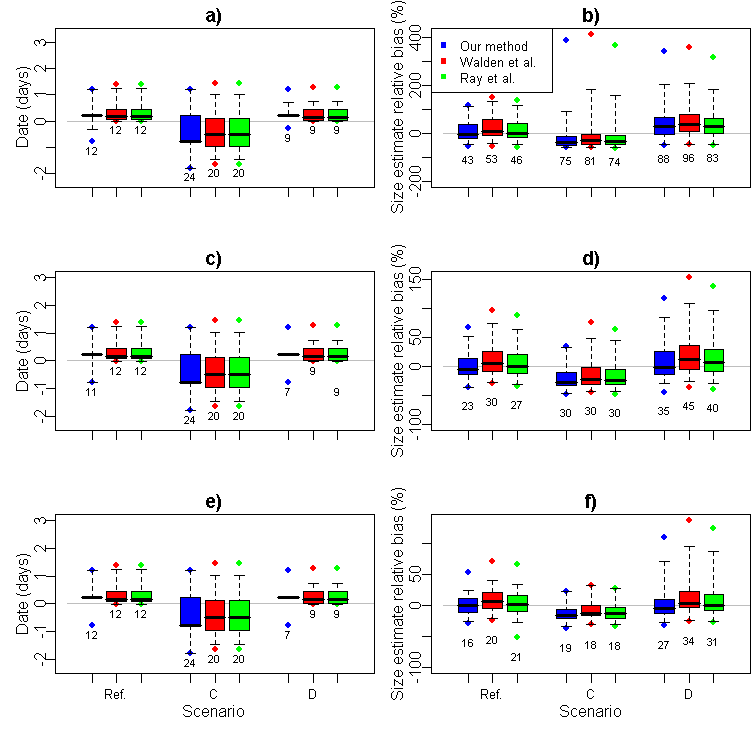


Figure S11: Number of simulations for which people living or working in a given ward would be missed by targeted controls (red dots) or inaccurately targeted (green dots).

**The size of each red/green dot correspond to the number of simulations for which the estimated risk in the ward is under/over 1/100 000 but not the risk computed with real parameters. The downwind and crosswind distance are indicated in km.**


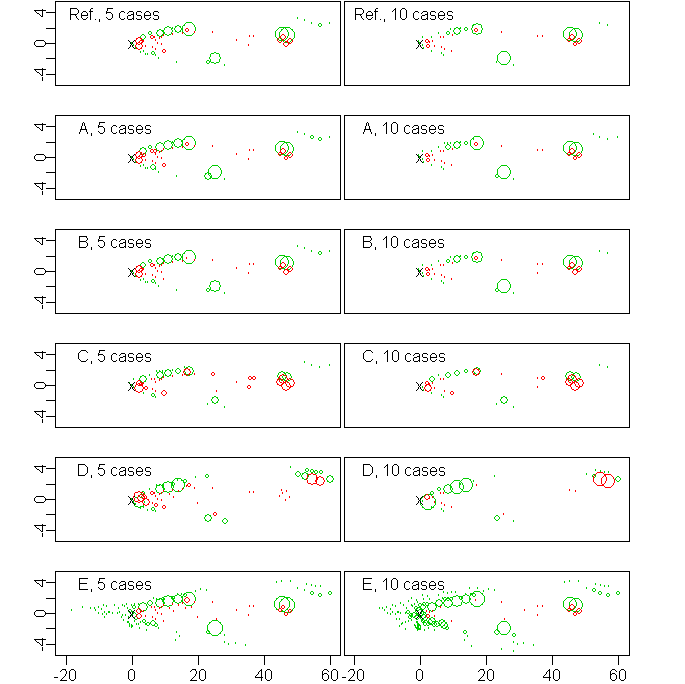


Figure S12: Impact of administering treatment to individuals living or working in a ward exposed to a given risk (reference scenario): outbreak size when there is no treatment (grey box-plots) and when a 100% effective prophylactic treatment is administered 4 days after the first 5 (a), 10 (b) or 15 (c) cases occurred.

**Each box-plot represents the distribution (minimum, maximum, percentiles 2.5, 25, 50, 75, 97.5) of the total number of cases.**


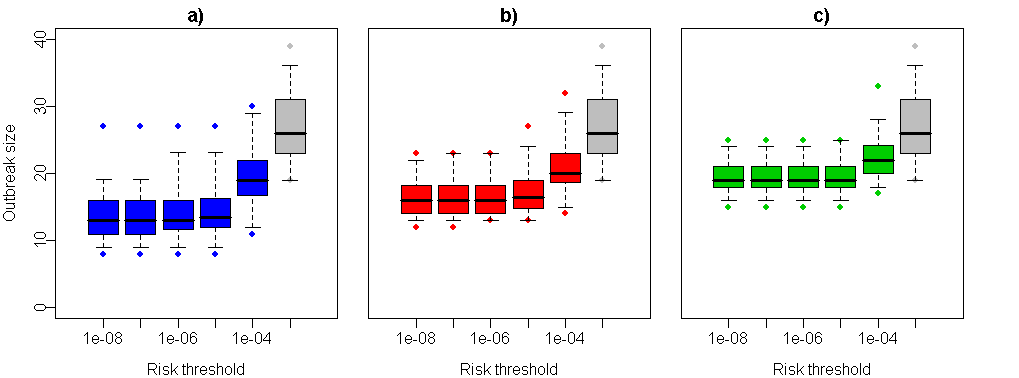


Figure S13: Comparison of the outbreak size when the mitigation strategy based on our estimates was applied and when a ring strategy around the 3 most infected wards (J=3) was applied.

#
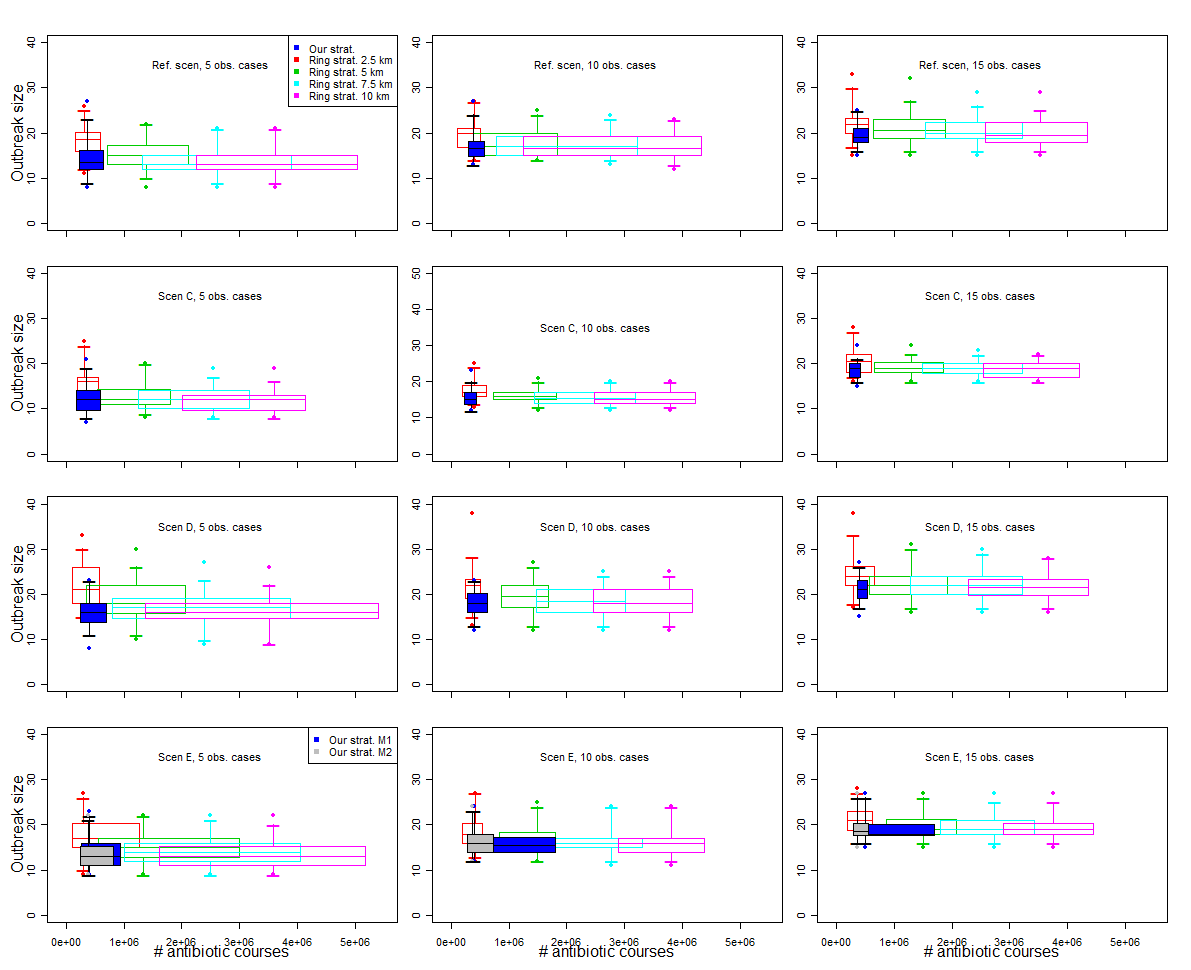


# REFERENCES

1. Seinfeld JH (1986) Atmospheric chemistry and physics of air pollution. New York: Wiley&sons.

2. Wein LM, Craft DL, Kaplan EH (2003) Emergency response to an anthrax attack. Proc Natl Acad Sci U S A 100: 4346-4351.

3. Stuart AL, Wilkening DA (2005) Degradation of biological weapons agents in the environment: implications for terrorism response. Environ Sci Technol 39: 2736-2743.

4. Brookmeyer R, Johnson E, Barry S (2005) Modelling the incubation period of anthrax. Stat Med 24: 531-542.

5. Bailey NTJ (1990) The Elements of Stochastic Processes with Applications to the Natural Sciences. New York: Wiley.

6. Wilkening DA (2006) Sverdlovsk revisited: modeling human inhalation anthrax. Proc Natl Acad Sci U S A 103: 7589-7594.

7. Cauchemez S, Valleron AJ, Boelle PY, Flahault A, Ferguson NM (2008) Estimating the impact of school closure on influenza transmission from Sentinel data. Nature 452: 750-754.

8. Lee BL (2005) The Profile Sampler. Journal of the American Statistical Association 100: 960.

9. Murphy SA, van der Vaart AW (2000) On Profile Likelihood. Journal of the American Statistical Association 95: 449-465.
